# Aging as a multifactorial disorder with two stages

**DOI:** 10.18632/aging.206339

**Published:** 2025-12-30

**Authors:** David Gems, Alexander Carver, Yuan Zhao

**Affiliations:** 1 Institute of Healthy Ageing, and Research Department of Genetics, Evolution and Environment, University College London, London WC1E 6BT, UK; 2 School of Biological and Behavioural Sciences, Queen Mary University of London, E1 4DQ, UK

**Keywords:** aging, *C. elegans*, disease, hyperfunction, multifactorial model

## Abstract

Aging (senescence) is characterized by development of diverse senescent pathologies and diseases, leading eventually to death. The major diseases of aging, including cardiovascular disease, cancer and chronic obstructive pulmonary disease (COPD), are multifactorial disorders, resulting from complex interactions between multiple etiologies. Here we propose a general account of how different determinants of aging can interact to generate late-life disease. This account, initially drawn from studies of the nematode *Caenorhabditis elegans*, depicts senescence as the product of a two-stage process. The first stage involves the diverse causes of disease prior to aging, that cause disruption of normal biological function. These include infection, mechanical injury and mutation (somatic and inherited). Second, etiologies largely confined to aging: deleterious, late-life consequences of evolved wild-type gene action, including antagonistic pleiotropy. Prior to aging, diverse insults lead to accumulation of various forms of injury that is largely contained, preventing progression to major pathology. In later life, wild-type gene action causes loss of containment of latent disruptions, which form foci for pathology development. Pathologies discussed here include osteoarthritis, cancer, late-life recrudescence of infection, and consequences of late-acting deleterious mutations. Such latent injury foci are analogous to seeds which in later life, in the context of programmatic senescent changes, germinate and develop into disease.

## INTRODUCTION

This article is a contribution to the special issue of Aging celebrating the life and work of Misha Blagosklonny (more formally, Mikhail Vladimirovich Blagosklonny), who died in October 2024 [1]. Here we will first briefly introduce several of his key contributions to aging theory, that opened the way to further conceptual developments. We will then describe in detail some new ideas that build upon his earlier breakthroughs.

**Figure 1:**
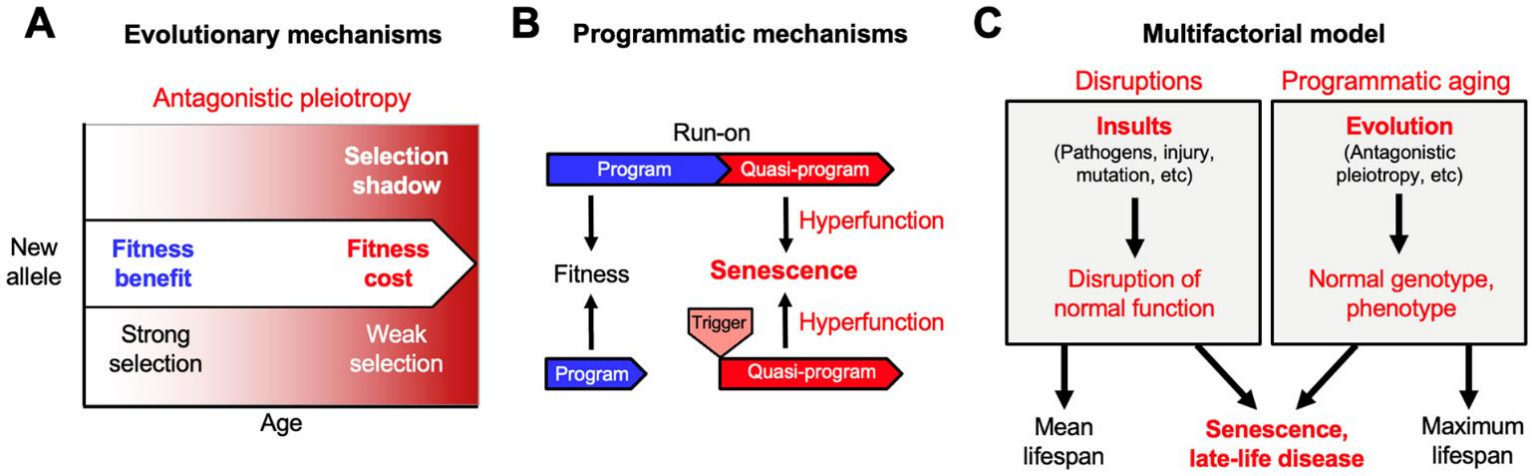
**From the evolutionary theory to the multifactorial model.** Developing new theory from foundations laid by Misha Blagosklonny. (**A**) The evolutionary theory of aging. Selection declines at later ages (selection shadow). Consequently, new alleles that promote fitness earlier in life but cause pathology at later ages may be favored by natural selection (antagonistic pleiotropy, AP). (**B**) Quasi-programmed hyperfunction. AP can be expressed as quasi-programs: genetically determined but non-adaptive derivatives of programs that promote fitness earlier in life. These may arise due to futile program run-on (top) as originally envisaged by Blagosklonny [18], or triggered reactivation (bottom) [23]. (**C**) The multifactorial model, in simplified form; a more detailed version was presented previously [22]. Late-life diseases are typically multifactorial in etiology. These etiologies fall into two broad categories: insults leading to disruption of normal biological function (left, e.g. infectious pathogens, mechanical injury, mutation); and programmatic action of the normal genotype (**B**), that are pathogenic due to the evolutionary process (**A**). Differences in lifespan between individuals, and mean lifespans of populations, are strongly determined by disruptions. Maximum (and also mean) lifespan is determined by programmatic aging, as specified by the normal genome. For example, the shorter maximum lifespan of the mouse *Mus musculus* (4 years) than the naked mole rat *Heterocephalus glaber* (31 years) is determined by the normal genome, not disruptions.

The community of scientists investigating the biology of aging are brought together more by their interest in the subject than by any shared ideas about its causes. To this day the field has relatively little consensus with respect to basic, foundational principles. However, it has long been argued that such a foundation may be found in the evolutionary theory of aging [2, 3]. From the very great difference in aging rate and maximum lifespan across animal species it is evident that senescence is predominantly genetically determined, and therefore the product of evolution. For more than half a century, the evolutionary theory of aging has provided a cogent explanation for the existence of aging [4, 5]. Most fundamentally, this argues that aging evolves due to reduced selection in later life - the so-called selection shadow (Figure 1A). This tells us that aging is not an adaptation.

The selection shadow can lead to accumulation in populations of mutations with harmful effects in later life, that contribute to aging - this is Peter Medawar’s mutation accumulation (MA) theory [5]. Aging can also evolve as a consequence of the pleiotropic nature of many genes, i.e. where they affect more than one biological characteristic. In some cases, genes have both beneficial and harmful consequences; in other words, pleiotropy has antagonistic effects, on health or fitness. As noted by George C. Williams, if the beneficial effect of a particular allele (gene variant) is experienced earlier, and the detrimental effect late enough, natural selection may favor that allele, increasing both fitness and aging [4] (Figure 1A). The relative importance of antagonistic pleiotropy (AP) and MA remains a topic of debate [6, 7]; however, numerous genes exhibit AP [8, 9].

A long-standing problem is that the evolutionary theory explains why we age but not how we age. It tells us that genes cause aging, and that this can involve MA and AP, but not how genes cause aging, including late-life disease. We lack an explanation in terms of evolutionary physiology [3], that unites ultimate (evolutionary) and proximate (mechanistic) accounts.

An important development in theories of aging was the appearance in the 1970s of an evolutionary physiology account, the disposable soma theory [10, 11]. This reconciled with evolutionary theory the then predominant view that aging is caused by molecular damage accumulation. It argued that processes of somatic maintenance that prevent aging are costly in resource terms, and that such processes compete with growth and reproduction for limited resources. Because natural selection prioritises reproduction over longevity, investment into somatic maintenance is inadequate to prevent aging, i.e. the soma is disposable, and akin to a cheaply-manufactured, throw-away consumer product.

However, research during the 2000s increasingly challenged the view that molecular damage is the main, primary cause of aging, casting doubt on the disposable soma theory [12–14]. Moreover, a major role of disposable soma-type mechanisms in lifespan-reproduction trade-offs remains undemonstrated, and in some cases, they clearly do not occur [15–17]. In the mid-2000s an alternative evolutionary physiology account was proposed, by Blagosklonny [12, 18, 19] and also João Pedro de Magalhães [20, 21].

For a detailed overview of this alternative account, see [22]. More briefly: echoing an earlier interpretation of AP by Williams himself [4], it argued simply that gene action in later life is pathogenic. Genetically-determined programs that contribute to fitness may in later-life run on, or be reactivated, in a manner that promotes pathology (Figure 1B). Blagosklonny noted that such gene action is genetically determined (programmed) but not adaptive (not programmed); to describe this, and disambiguate these two meanings of the word programmed, he introduced the term quasi-programmed [18]. He argued that aging is far from being a process of passive breakdown (as in molecular damage accumulation), of loss of function, but rather is the opposite: an active, quasi-programmed process, of hyperfunction. Hence this evolutionary physiology account is sometimes referred to as the hyperfunction theory. Blagosklonny himself also referred to it as disposable soma theory 2 since the soma is still disposable, though due to quasi-program action rather than insufficient somatic maintenance [19, 24].

Arguably, the importance of the hyperfunction theory is that it provides a new starting point and direction for thinking about evolutionary physiology, and proximate mechanisms of aging. Further development of programmatic theory beyond hyperfunction include concepts such as hypofunction [17, 20], generation of AP as bad spandrels by biological constraint [9], costly programs in the context of semelparous reproductive death [25–27], and adaptive death [28–30], among other ideas [31–33], plus several demonstrations of the action of quasi-programs in aging *C. elegans* in the Gems lab [34–36].

## The Multifunctional Model

A further elaboration of the hyperfunction theory, proposed by one of us in 2022, is the multifactorial model [22]. One reason why Blagosklonny argued against the molecular damage theory is its inadequacy as an explanation for diseases of aging (with the exception of cancer). By contrast, he showed how hyperfunction is identifiable as part of the etiology of a range of late-life diseases [18, 22]. The multifactorial model represents an attempt to improve on the explanatory power of the hyperfunction theory with respect to late-life disease. The origin of the model is experimental work using *C. elegans* (described below), and also the four-models theory developed in the 1990s by Vladimir Dilman [22, 37]; for more on Dilman’s contributions to biogerontology, see [38].

For a detailed account of the multifactorial model, see [22]. Figure 1C presents a simplified version of it. A feature of diseases of aging familiar to clinicians is that they are highly multifactorial in etiology; this includes cardiovascular disease, COPD, Alzheimer's disease, osteoarthritis, and osteoporosis [39–44]. The multifactorial model argues that these etiologies, very broadly, fall into two categories.

The first is etiologies involving insults that cause disease by disrupting normal biological function. Such insults include infectious pathogens (e.g. viruses, bacteria, protozoa, helminths), mechanical injury, mutation (somatic and inherited), and poor nutrition. Disruptions are represented by the left-hand box in Figure 1C. The second category of etiology includes pathogenic, programmatic changes that occur in later life, as specified by the wild-type genome. These can be understood as, ultimately, the consequence of evolution. It includes late-life effects of AP, that do not result from insults, but from wild-type gene action [45]. This is represented by the right box in Figure 1C.

The multifactorial model views senescence, including diseases of aging, as the outcome of both disruptions and programmatic aging. According to this view, disruptions are very much part of senescence. Moreover, some diseases that are usually viewed as disruptions are very much diseases of aging. To illustrate, consider sudden acute respiratory syndrome (SARS), a major cause of death during the SARS-CoV-2 (COVID-19) pandemic. There SARS occurred mainly among the elderly, due to age changes in immunity, particularly hyperactivity of innate immune function, of programmatic origin [46]. Due to such changes, infections with coronavirus could trigger a lethal cytokine storm, leading to SARS and death. Thus, SARS triggered by SARS-CoV-2 typifies multifactorial diseases of aging. Consistent with this view, age-specific death rates from many causes traditionally viewed as extrinsic increase exponentially with age [47].

In the remainder of this article we will describe further features of the multifactorial model, particularly the relationship between disruptions that occur in early life, and the later processes of programmatic senescence.

## How distinct causes of senescence interact: a two-stage model

The distinction between disruptions and genetically-determined etiologies in the multifactorial model (Figure 1C) raises the question of how these two classes of cause interact. Here two plausible premises for further conjecture are (i) that disruptions, since they occur throughout life, will occur prior to the appearance of programmatic aging; and (ii) that, in the main, only through interaction with programmatic changes will disruptions give rise to senescent changes.

**Figure 2:**
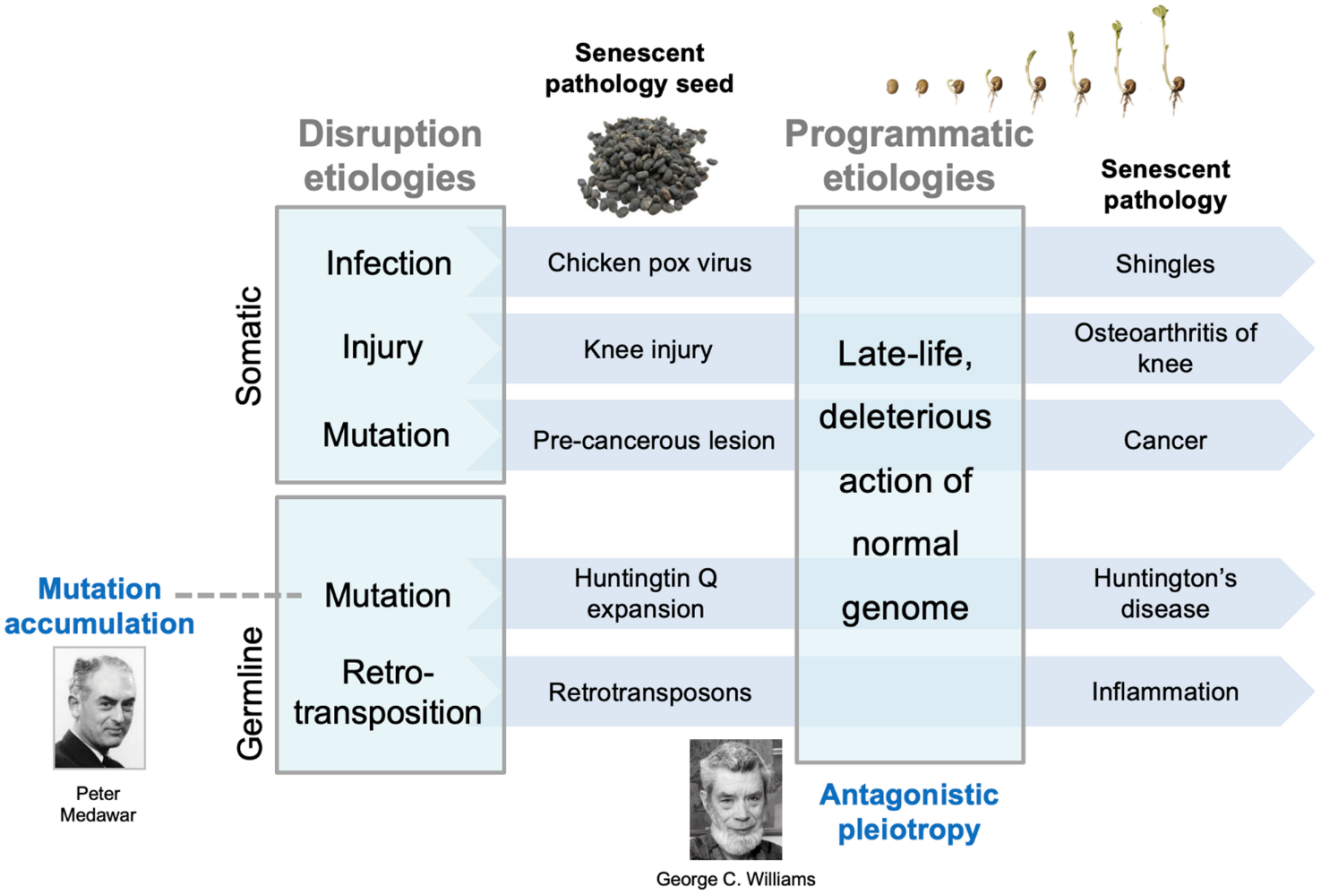
**Two-stage model for interactions between earlier and later etiologies.** Diverse disruptions of normal biological function resulting from insults are contained and lie dormant within the youthful physiological milieu. In the senescent milieu, containment of such disruptions fails, and they form foci for the development of diverse senescent pathologies. Such contained, latent disruptions are analogous to seeds lying dormant within the host; later, pathogenic wild-type gene action stimulates the seeds to grow; this analogy captures the developmental nature of senescent pathogenesis [20]. The early etiologies are those typical of disease causes prior to aging, including infection, mechanical damage, and mutation (somatic and inherited). The main, late-life etiology, wild-type gene action, is predominantly (but not entirely) restricted to senescence. The model encompasses both evolutionary theories of aging. Mutation accumulation (MA): inherited, late-acting deleterious mutations can be understood as those unmasked by later programmatic changes. Antagonistic pleiotropy (AP): this determines the late-life programmatic changes themselves. Note that this model does not argue against a role for molecular damage accumulation in aging, but rather that it is a relatively minor contributory factor (e.g. DNA damage in cancer development).

Here we present a two-stage model for the development of diseases of aging. This involves two main stages, an early stage involving disruptions only, and a later stage involving both disruptions and programmatic changes. After the diverse insults sustained prior to senescence, the consequent disruptions are often eliminated and health fully restored. However, some disruptions are not fully resolved and are instead contained, allowing health to be sustained during development and reproduction. Critically, latent injury re-emerges as a consequence of programmatic changes caused by the wild-type genome, and ultimately the evolutionary process. These recrudescent injuries form foci for the development of disease (Figure 2).

Using an analogy to illustrate the model: the latent, disruption-derived injuries are akin to seeds that remain dormant during early adulthood and midlife; in later life, processes of senescence determined by the wild-type genome cause the seeds to germinate into late-life pathologies (Figure 2). An alternative perspective, that emphasises the action of programmatic mechanisms in pathogenesis, is that only in later life can disruptions trigger the development of programmatic pathologies [48].

## Origins of the model: senescence of the *C. elegans* pharynx

**Figure 3:**
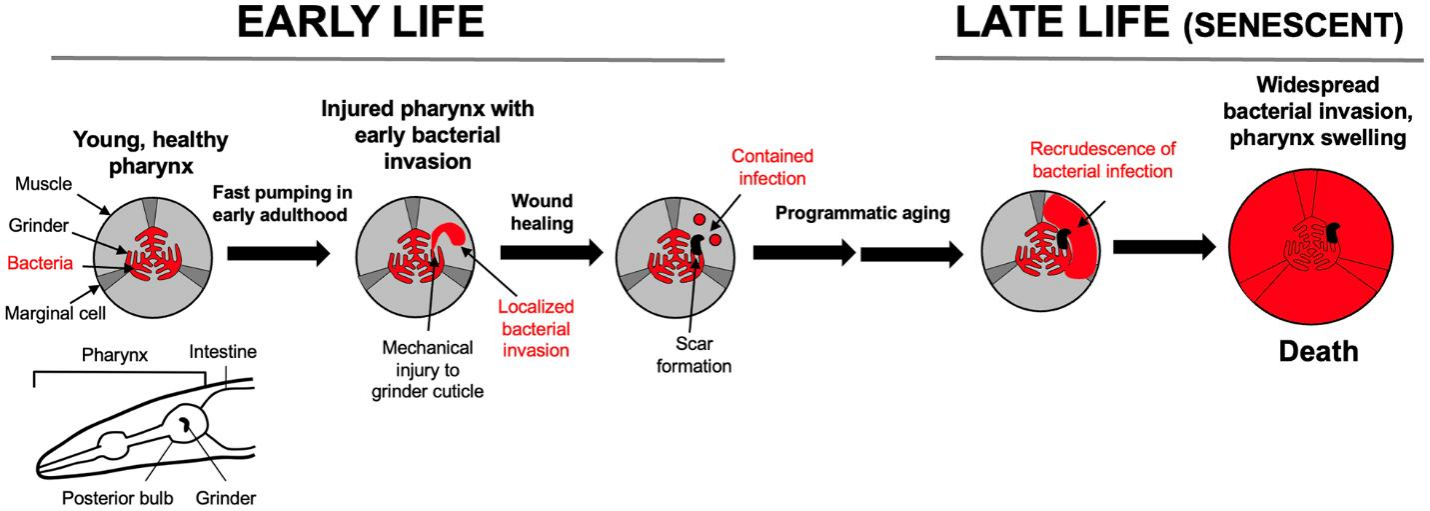
**Pathophysiology of pharyngeal senescence in aging *C. elegans*.** High rate of pharyngeal pumping in early adulthood leads to mechanical damage (mechanical senescence) to the pharyngeal cuticle. This perforates the cuticle, allowing minor bacterial invasion of pharyngeal tissue. In later life in ~40% of individuals, the invasion spreads further, leading to widespread infection, pharyngeal swelling and death [49]. Bottom left: schematic representation of *C. elegans* anterior end (head), including the pharynx. A caveat here is that laboratory culture of *C. elegans* with *E. coli* is not necessarily representative of the experience of this organism in the wild.

We first conceived of the multifactorial model after studying the origins of late-life disease in *C. elegans*. Under standard laboratory culture conditions, a common cause of death in aging *C. elegans* involves infection of the pharynx, the muscular tube anterior to the intestine which ingests and grinds up the worm’s microbial food source (in laboratory studies, *Escherichia coli* OP50) [49]. Emergence of this pathology involves at least three distinct etiological components (Figure 3).

First, in early adulthood the rapid chewing action of the pharyngeal grinder leads to perforation of the cuticle that lines the pharynx, due to mechanical damage, i.e. mechanical senescence [50]. Second, small numbers of *E. coli* invade pharyngeal tissue via the cuticular perforations. These bacilli are then sequestered into membrane-bound inclusions within the cells of the pharynx, probably aided by host innate immunity. The cuticular perforations subsequently heal, leaving a latent infection within the pharynx. In later life, the contained bacilli re-emerge, proliferate and develop into major infections that destroy pharyngeal tissue, causing swelling of the pharynx and, shortly thereafter, death [49]. A plausible cause of this later expansion of infection is programmatic aging resulting from the wild-type genome, including immunosenescence [51, 52].

Thus, according to our working model, early injury due to two etiologies, mechanical injury and infection, are contained, but in later life wild-type gene action unmasks the latter, leading to progression to major pathology and death. We subsequently used a conceptual research approach [53], exploring existing literature to test the relevance of this two-stage model to late-life disease in humans, beginning with late-life infection.

## Senescent recrudescence of early life infection

The pattern seen in the etiology of *C. elegans* death with pharyngeal infection resembles the phenomenon of senescent recrudescence (i.e. reappearance) of infection in humans. This can occur in situations where infectious pathogens are largely cleared earlier in life, but where residual infection escapes host immunity. Such latent infection can re-emerge at times of host immune deficiency, for example due to immunosenescence.

An example of this is chicken pox in childhood remerging as shingles during aging. Chicken pox is caused by infection with the *Varicella zoster* virus (HZV), which is highly prevalent among children, e.g. 77% by the age of 5 in the UK [54]. The primary infection is characterised by lesions on the skin which normally clear within two weeks of first appearance [55]. For a small number of patients, this primary infection develops into shingles (herpes zoster), typically many years or decades after the original infection. The symptoms of shingles differ markedly from those of chicken pox, typically presenting as a vesicular rash (i.e. with fluid-filled sacs) isolated to one dermatome (area of skin provided by a single spinal nerve), and often accompanied by severe pain [56].

*V. zoster* virions are thought to remain dormant within the dorsal ganglia, re-emerging at times of immune suppression, particularly due to aging. Incidence of shingles increases strongly with advancing age [57–59]. For example, one UK study reported an age increase in annual shingles incidence from 0.74 per 1,000 in 0–9-year-olds to 10.1 in 0–89-year-olds [58]. This likely reflects the decline in immune function in late life.

*V. zoster* is one of a number of pathogens showing senescent recrudescence. Others include polio, caused by Poliovirus, which recurs as post-polio syndrome [60–62]; typhus, caused by the bacterium *Rickettsia prowazekii*, which recurs as Brill-Zinsser Disease [63–66]; and also tuberculosis (TB), caused by the bacterium *Mycobacterium tuberculosis*.

According to a World Health Organization estimate, around a quarter of the world population is infected with *M. tuberculosis*, but in most cases, infections are latent and asymptomatic (latent TB infection, LTBI). In LTBI, mycobacteria in the lungs are contained and proliferation controlled within granulomas, clusters of phagocytic leukocytes (neutrophils, macrophages), surrounded by T and B lymphocytes. In the progression of LTBI to the more severe, active form of TB, mycobacteria break free from granulomas. Once mycobacteria have spread throughout the lungs, the infection is considered active and can be spread by droplet transmission. Active TB presents with symptoms ranging from those that resemble flu, to multiple organ failure [67].

Various factors can increase the relative risk of progression from LTBI to active TB, including surgery, drug abuse, HIV, and also advancing age [68–70]. For example, one study found that the incidence rate per 100,000 people rose from 7.3 at ages 21-64 to 14.2 at ages 85-94 [70]. In each of these examples, as with contained early infections in the *C. elegans* pharynx, latent infections recrudesce during organismal senescence, leading to disease.

## Early mechanical injury as a focus for late-life disease

The first in the series of etiologies leading in *C. elegans* to death with an infected pharynx is mechanical injury. Can mechanical injury in early life seed senescent pathology in humans? The *C. elegans* grinder is one of the few, hard moving parts in this organism; by contrast, in the vertebrate skeleton these are numerous, in the form of articular joints. Mechanical wear and tear to joints is one of a number of etiologies contributing to osteoarthritis (OA), age-related degeneration of cartilage and bone. A common joint for disabling OA is the knee, with 13% of women and 10% of men over the age of 60 suffering from symptomatic knee OA.

Risk factors for OA include age, female sex, obesity and repetitive joint trauma [71]. Notably, earlier life joint injury increases the risk in old age of OA of the knee [72–75] and also the hip [76]. For example, one Finnish study controlling for other covariates, including age and sex, estimated an adjusted odds ratio of 5.1 for previous knee injury in people with knee OA. It remains unclear whether OA emerges due to later senescent changes causing earlier joint injury to become “uncontained”, or whether subtle changes in gait after injury cause wear-and-tear changes to occur more rapidly.

Nonetheless, the two-stage model provides a possible account of interactions between etiologies contributing to OA. The first stage occurs due to mechanical insults to joints, to which various inherited or congenital factors may predispose. The second involves wild-type gene action and programmatic changes, leading to gradual change and hyperfunction in chondrocytes in particular [48]. More broadly, the relationship between earlier joint injury and later OA is consistent with the two-stage model (Figure 3).

Another form of disruption caused by mechanical stress is traumatic brain injury (TBI), resulting from head injury. Individuals from professions with increased exposure to head trauma, including boxers, American football players and soldiers, have increased risk of chronic traumatic encephalopathy in later life [77–80]. In the general population, head trauma in early life is associated with an increased risk of developing other forms of late-life dementia [81, 82]. For example, a Taiwanese study of a cohort of one million people, followed between 2005 and 2009, found that 28,551 patients suffered from mild traumatic brain injury (mTBI) [82]. For those with mTBI the incidence rate of dementia was 1.8 per 1,000 person-year compared to only 0.3 per 1,000 person-year in those without mTBI (hazard ratio 6.34). The nature of the latent damage after TBI is unclear, but one possibility is altered function of the glymphatic system [83].

## Early somatic mutation and late-life cancer

Another form of disruption leading to disease is somatic mutation, mainly as a cause of cancer. The largest risk factor for cancer is advancing age, and cancer incidence increases exponentially with age [84, 85]. The age increase in cancer rate was initially attributed to time-dependent somatic mutation accumulation alone, as described by the multi-stage theory of carcinogenesis [86]. More recently, evidence has emerged that senescent changes, particularly to the microenvironment of pre-neoplastic cells, increases the likelihood of progression to metastatic cancer. One predicted driver of such changes is accumulation of senescent cells (sensu Hayflick), including senescent fibroblasts [87, 88].

Fibroblast senescence was originally described as cell proliferative exhaustion *in vitro*, due to shortening of telomeres, and understood as a tumor suppressor mechanism [89]. However, after leaving the cell cycle fibroblasts undergo major differentiative changes (geroconversion), becoming hypertrophic and hyper-secretory, exhibiting the senescence-associated secretory phenotype (SASP), which promotes development of tumors [90] and, as suggested by murine studies, many other senescent pathologies [91, 92]. This mysterious behavior in senescent fibroblasts was explained by the discovery that senescent fibroblasts contribute to wound healing [93]. Thus, the pathogenic action of senescent cells appears to result from quasi-programmed recapitulation of tissue-remodelling and inflammatory functions [18, 94]. The interaction between somatic mutations and senescent cell accumulation conforms to the two-stage model (Figure 3). The first etiology involves disruption (DNA damage), and the second programmatic changes leading to futile activation of tissue-remodelling functions. Currently, the cause of senescent cell accumulation *in vivo* is unclear [95]; the two-stage model postulates that it is largely programmatic.

A related phenomenon is late-life recurrence of cancer that was successfully treated in early life, which can occur, for example, with breast cancer [96–98]. For instance, in one case a 73-year-old woman presented with a secondary carcinoma of the breast 35 years after the original breast carcinoma was removed [96]. The authors of the report suggested that cells from the original carcinoma had lain dormant and, due to age changes in the patient, had subsequently developed into the secondary carcinoma.

## Inherited mutations and late-life disease

As described, the mutation accumulation theory argues that the selection shadow leads to accumulation in populations of inherited mutations with late-life deleterious effects [5]. J.B.S. Haldane reasoned that this could explain the relatively high prevalence of Huntington's disease, despite its genetically dominant mode of inheritance: because the neurodegenerative symptoms emerge only in mid-life, the force of selection to eliminate the mutation is relatively weak [99]. Many genetic diseases show mid- to late-life onset, often accelerating one or more senescent pathology (unimodal or segmental progeroid conditions, respectively) [100]. For example, mutations in various genes linked to β-amyloid processing lead to early onset (familial) Alzheimer's disease [101], and mutation of the protein component of telomerase (TERT) leads to increased levels of idiopathic pulmonary fibrosis (IPF) [102].

But what, in terms of biological mechanisms, is a late-acting deleterious mutation? How do the harmful effects of such mutations remain latent, and then break out in late life to cause pathology? The two-stage model provides a potential explanation. We suggest that, as with other forms of disruption-related etiology, harmful effects of such mutations are unmasked by interactions with late-life changes that are, in particular, driven by late-life gene action (Figure 3). For example, senescent cell accumulation and SASP production, which promotes IPF [103], and is at least partly programmatic in origin [94], could interact with TERT mutations to cause IPF. Thus, in the two stage model disruptions encompass not only those experienced early in life, but also those affecting the ancestral genome in its recent and distant past.

The principle involved here is somewhat akin to the classic 1998 study of Hsp90 deficiency in *Drosophila* where, due to impaired protein folding homeostasis, mutant effects of diverse genetic variants were unmasked [104]. The difference here is that in aging it is programmatic changes (rather than loss of proteostasis) that lead to such unmasking effects, increasing penetrance of late-acting deleterious mutations. For Huntington’s there is evidence that age changes unmask the mutant phenotype of the causative mutation [105, 106].

## Retrotransposon activation

One major, inherited genetic disruption is the large number of retrotransposon-derived sequences, that make up ~45% of the human genome [107]. During youth and early adulthood, these retrotransposons remain silent, but during organismal aging, and also cellular senescence *in vitro*, a small proportion become active in a “jail break” mechanism [108, 109]. The awakening of these “sleeping dogs of the genome” [110] leads to further DNA damage, including insertional mutagenesis. This illustrates again the principle of early containment and later escape of disruption (here inherited) (Figure 2).

When retrotransposon (L1) de-containment occurs during late cellular senescence, this triggers gene expression changes, including a type 1 interferon response that promotes sterile inflammation [111]. Here during cellular senescence, wild-type gene action promotes de-containment of disruption-derived injury, leading to further, pathogenic wild-type gene action. By contrast, one driver of de-containment during aging is accumulation of double-strand DNA breaks [112]. This leads to changes in histone methylation and acetylation involving redistribution of the histone deacetylase SIRT6 [113]. Here, damage accumulation leads to de-containment of other disruptions (retrotransposons), leading to further disruption and programmatic change.

## Final Remarks

The multifactorial model presents a complex account of the causes of aging, encompassing the evolutionary theory, disruptions and programmatic mechanisms. Here we derive from it a two-stage model that offers an understanding of the origins of some (though doubtless not all) late-life diseases. Given that most late-life diseases are multifactorial disorders, it is no wonder that aging is so difficult to understand. The researcher of aging is akin to a doctor struggling to make a diagnosis with a patient suffering from, say, hepatitis C, toxocariasis, mercury poisoning and Klippel-Trenaunay syndrome, and their complex interactions.

## The multifactorial model is an evolutionary physiology account

Like the more purely programmatic accounts of Williams, Blagosklonny and de Magalhães [4, 12, 18, 20], the multifactorial model provides an evolutionary physiology perspective. It argues that natural selection maintains fitness until after the onset of reproduction, dealing with disruptions either by resolving or, failing that, containing them. A consequence of declining selection at later ages is loss of containment of such disruptions. This contributes to late-life disease, as described for osteoarthritis, cancer, recrudescence of infection, and manifestation of genetic diseases with late onset, including unimodal and segmental progeroid conditions.

## The multifactorial model and anti-aging treatments

Better definition of aging and its causes allows a clearer conception of treatments for aging. Anti-aging treatments can be understood as interventions which counteract any etiology of organismal senescence [114]. The multifactorial model defines two basic types of anti-aging treatment. First, those that prevent causes of disruption. This could include prevention of infection (e.g. immunization against *V. zoster*, preventing shingles, and also Alzheimer’s disease [115]), of mechanical injury (e.g. use of protective gear in contact sports), and of mutation, both somatic (e.g. sunblock to avoid mutation of cells in skin, cleaner air measures), and inherited (pre-implantation genetic screening, and perhaps one day CRISPR-CAS9 correction of mutations). Second, those that suppress pathogenic late-life, wild-type gene action. One example here is use of rapamycin to inhibit mTOR-driven hyperfunction and quasi-programs (e.g. SASP production), a possibility much discussed by Blagosklonny [22]. Rapamycin has robust anti-aging effects in mice, extending healthspan and lifespan [116, 117], but whether it similarly affects humans is far from clear. Thus, at present, the available anti-aging treatments with efficacy in human beings are those that act by preventing disruptions.

## References

[1] Gems D, Demaria M. Misha Blagosklonny: A life of ideas. Aging. 2025; In press. 10.18632/aging.206412 42565761

[2] Rose MR. Evolutionary Biology of Aging. Oxford: Oxford University Press. 1991.

[3] Arnold KR, Rose MR. Conceptual Breakthroughs in The Evolutionary Biology of Aging. Academic Press. 2023.

[4] Williams GC. Pleiotropy, natural selection and the evolution of senescence. Evolution. 1957; 11:398-411.

[5] Medawar PB. An Unsolved Problem Of Biology. London: H.K. Lewis. 1952

[6] Flatt T, Partridge L. Horizons in the evolution of aging. BMC Biol. 2018; 16:93. 10.1186/s12915-018-0562-z 30124168 PMC6100731

[7] Zhao X, Promislow DEL. Senescence and ageing. In The Oxford Handbook of Evolutionary Medicine, M. Brüne and W. Schiefenhövel, eds. Oxford, UK: Oxford University Press, 2019. pp. 167–208.

[8] Austad SN, Hoffman JM. Is antagonistic pleiotropy ubiquitous in aging biology? Evol Med Public Health. 2018; 2018:287–94. 10.1093/emph/eoy033 30524730 PMC6276058

[9] Gems D, Kern CC. Biological constraint, evolutionary spandrels and antagonistic pleiotropy. Ageing Res Rev. 2024; 101:102527. 10.1016/j.arr.2024.102527 39374830 PMC7618566

[10] Kirkwood TB. Evolution of ageing. Nature. 1977; 270:301–4. 10.1038/270301a0 593350

[11] Kirkwood TB, Rose MR. Evolution of senescence: late survival sacrificed for reproduction. Philos Trans R Soc Lond B Biol Sci. 1991; 332:15–24. 10.1098/rstb.1991.0028 1677205

[12] Blagosklonny MV. Aging: ROS or TOR. Cell Cycle. 2008; 7:3344–54. 18971624 10.4161/cc.7.21.6965

[13] Pérez VI, Bokov A, Van Remmen H, Mele J, Ran Q, Ikeno Y, Richardson A. Is the oxidative stress theory of aging dead? Biochim Biophys Acta. 2009; 1790:1005–14. 10.1016/j.bbagen.2009.06.003 19524016 PMC2789432

[14] Gems D, Doonan R. Antioxidant defense and aging in C. elegans: is the oxidative damage theory of aging wrong? Cell Cycle. 2009; 8:1681–7. 10.4161/cc.8.11.8595 19411855

[15] Grandison RC, Piper MD, Partridge L. Amino-acid imbalance explains extension of lifespan by dietary restriction in Drosophila. Nature. 2009; 462:1061–4. 10.1038/nature08619 19956092 PMC2798000

[16] Zajitschek F, Georgolopoulos G, Vourlou A, Ericsson M, Zajitschek SR, Friberg U, Maklakov AA. Evolution Under Dietary Restriction Decouples Survival From Fecundity in Drosophila melanogaster Females. J Gerontol A Biol Sci Med Sci. 2019; 74:1542–8. 10.1093/gerona/gly070 29718269

[17] Maklakov AA, Chapman T. Evolution of ageing as a tangle of trade-offs: energy versus function. Proc Biol Sci. 2019; 286:20191604. 10.1098/rspb.2019.1604 31530150 PMC6784717

[18] Blagosklonny MV. Aging and immortality: quasi-programmed senescence and its pharmacologic inhibition. Cell Cycle. 2006; 5:2087–102. 10.4161/cc.5.18.3288 17012837

[19] Blagosklonny MV. Paradoxes of aging. Cell Cycle. 2007; 6:2997–3003. 10.4161/cc.6.24.5124 18156807

[20] de Magalhães JP, Church GM. Genomes optimize reproduction: aging as a consequence of the developmental program. Physiology (Bethesda). 2005; 20:252–9. 10.1152/physiol.00010.2005 16024513

[21] de Magalhães JP. Programmatic features of aging originating in development: aging mechanisms beyond molecular damage? FASEB J. 2012; 26:4821–6. 10.1096/fj.12-210872 22964300 PMC3509060

[22] Gems D. The hyperfunction theory: An emerging paradigm for the biology of aging. Ageing Res Rev. 2022; 74:101557. 10.1016/j.arr.2021.101557 34990845 PMC7612201

[23] Kern C, Stebbing J. Uncovering the blueprint of aging: how aging causes late-life disease. Preprints.org. 2023. 10.20944/preprints202310.201387.v202312

[24] Blagosklonny MV. MTOR-driven quasi-programmed aging as a disposable soma theory: blind watchmaker vs. intelligent designer. Cell Cycle. 2013; 12:1842–7. 10.4161/cc.25062 23708516 PMC3735698

[25] Gems D, Kern CC, Nour J, Ezcurra M. Reproductive Suicide: Similar Mechanisms of Aging in C. elegans and Pacific Salmon. Front Cell Dev Biol. 2021; 9:688788. 10.3389/fcell.2021.688788 34513830 PMC8430333

[26] Kern CC, Townsend S, Salzmann A, Rendell NB, Taylor GW, Comisel RM, Foukas LC, Bähler J, Gems D. C. elegans feed yolk to their young in a form of primitive lactation. Nat Commun. 2021; 12:5801. 10.1038/s41467-021-25821-y 34611154 PMC8492707

[27] Kern CC, Srivastava S, Ezcurra M, Hsiung KC, Hui N, Townsend S, Maczik D, Zhang B, Tse V, Konstantellos V, Bähler J, Gems D. C. elegans ageing is accelerated by a self-destructive reproductive programme. Nat Commun. 2023; 14:4381. 10.1038/s41467-023-40088-1 37474586 PMC10359416

[28] Lohr JN, Galimov ER, Gems D. Does senescence promote fitness in Caenorhabditis elegans by causing death? Ageing Res Rev. 2019; 50:58–71. 10.1016/j.arr.2019.01.008 30639341 PMC6520499

[29] Galimov ER, Gems D. Shorter life and reduced fecundity can increase colony fitness in virtual Caenorhabditis elegans. Aging Cell. 2020; 19:e13141. 10.1111/acel.13141 32301222 PMC7253062

[30] Galimov ER, Gems D. Death happy: adaptive ageing and its evolution by kin selection in organisms with colonial ecology. Philos Trans R Soc Lond B Biol Sci. 2021; 376:20190730. 10.1098/rstb.2019.0730 33678027 PMC7938166

[31] Gems D. On Aging. What Causes It and How It Leads to the Maladies of Old Age. Columbia University Press. 2026.

[32] Lemaître JF, Moorad J, Gaillard JM, Maklakov AA, Nussey DH. A unified framework for evolutionary genetic and physiological theories of aging. PLoS Biol. 2024; 22:e3002513. 10.1371/journal.pbio.3002513 38412150 PMC10898761

[33] Slade L, Etheridge T, Szewczyk NJ. Consolidating multiple evolutionary theories of ageing suggests a need for new approaches to study genetic contributions to ageing decline. Ageing Res Rev. 2024; 100:102456. 10.1016/j.arr.2024.102456 39153601

[34] Wang H, Zhao Y, Ezcurra M, Benedetto A, Gilliat AF, Hellberg J, Ren Z, Galimov ER, Athigapanich T, Girstmair J, Telford MJ, Dolphin CT, Zhang Z, Gems D. A parthenogenetic quasi-program causes teratoma-like tumors during aging in wild-type C. elegans. NPJ Aging Mech Dis. 2018; 4:6. 10.1038/s41514-018-0025-3 29928508 PMC5998035

[35] Gems D, de la Guardia Y. Alternative Perspectives on Aging in Caenorhabditis elegans: Reactive Oxygen Species or Hyperfunction? Antioxid Redox Signal. 2013; 19:321–9. 10.1089/ars.2012.4840 22870907 PMC5395017

[36] de la Guardia Y, Gilliat AF, Hellberg J, Rennert P, Cabreiro F, Gems D. Run-on of germline apoptosis promotes gonad senescence in C. elegans. Oncotarget. 2016; 7:39082–96. 10.18632/oncotarget.9681 27256978 PMC5129915

[37] Dilman VM. An integrated, four-component mechanism of aging. In Development, Aging and Disease: A New Rationale for an Intervention Strategy. Harwood Academic Publishers, 1994. pp. 151–78.

[38] Golubev A. On the intergenerational transfer of ideas in aging and cancer research: From the hypothalamus according to V.M. Dilman to the mTOR protein complex according to M.V. Blagosklonny. Aging. 2025; In press. 10.18632/aging.206338 PMC12705181 41269205

[39] Glyn-Jones S, Palmer AJ, Agricola R, Price AJ, Vincent TL, Weinans H, Carr AJ. Osteoarthritis. Lancet. 2015; 386:376–87. 10.1016/S0140-6736(14)60802-3 25748615

[40] Glocker MO, Guthke R, Kekow J, Thiesen HJ. Rheumatoid arthritis, a complex multifactorial disease: on the way toward individualized medicine. Med Res Rev. 2006; 26:63–87. 10.1002/med.20045 16283676

[41] Al Anouti F, Taha Z, Shamim S, Khalaf K, Al Kaabi L, Alsafar H. An insight into the paradigms of osteoporosis: From genetics to biomechanics. Bone Rep. 2019; 11:100216. 10.1016/j.bonr.2019.100216 31372373 PMC6661363

[42] Higashi Y, Gautam S, Delafontaine P, Sukhanov S. IGF-1 and cardiovascular disease. Growth Horm IGF Res. 2019; 45:6–16. 10.1016/j.ghir.2019.01.002 30735831 PMC6504961

[43] Armstrong RA. What causes alzheimer's disease? Folia Neuropathol. 2013; 51:169–88. 10.5114/fn.2013.37702 24114635

[44] Huertas A, Palange P. COPD: a multifactorial systemic disease. Ther Adv Respir Dis. 2011; 5:217–24. 10.1177/1753465811400490 21429981

[45] Nesse RM, Williams GC. Why We Get Sick: The New Science of Darwinian Medicine. Random House. 1994

[46] Blagosklonny MV. From causes of aging to death from COVID-19. Aging (Albany NY). 2020; 12:10004–21. 10.18632/aging.103493 32534452 PMC7346074

[47] Koopman JJ, Wensink MJ, Rozing MP, van Bodegom D, Westendorp RG. Intrinsic and extrinsic mortality reunited. Exp Gerontol. 2015; 67:48–53. 10.1016/j.exger.2015.04.013 25916736

[48] Gems D. How aging causes osteoarthritis: An evolutionary physiology perspective. Osteoarthritis Cartilage. 2025; 33:921–32. 10.1016/j.joca.2025.05.001 40381687 PMC7618562

[49] Zhao Y, Gilliat AF, Ziehm M, Turmaine M, Wang H, Ezcurra M, Yang C, Phillips G, McBay D, Zhang WB, Partridge L, Pincus Z, Gems D. Two forms of death in ageing Caenorhabditis elegans. Nat Commun. 2017; 8:15458. 10.1038/ncomms15458 28534519 PMC5457527

[50] Comfort A. The Biology of Senescence. New York: Elsevier. 1979.

[51] Youngman MJ, Rogers ZN, Kim DH. A decline in p38 MAPK signaling underlies immunosenescence in Caenorhabditis elegans. PLoS Genet. 2011; 7:e1002082. 10.1371/journal.pgen.1002082 21625567 PMC3098197

[52] Lee Y, Jung Y, Jeong DE, Hwang W, Ham S, Park HH, Kwon S, Ashraf JM, Murphy CT, Lee SV. Reduced insulin/IGF1 signaling prevents immune aging via ZIP-10/bZIP-mediated feedforward loop. J Cell Biol. 2021; 220:e202006174. 10.1083/jcb.202006174 33666644 PMC7941181

[53] Blagosklonny MV, Pardee AB. Conceptual biology: unearthing the gems. Nature. 2002; 416:373. 10.1038/416373a 11919607

[54] Manikkavasagan G, Dezateux C, Wade A, Bedford H. The epidemiology of chickenpox in UK 5-year olds: an analysis to inform vaccine policy. Vaccine. 2010; 28:7699–705. 10.1016/j.vaccine.2010.09.017 20869468

[55] Mueller NH, Gilden DH, Cohrs RJ, Mahalingam R, Nagel MA. Varicella zoster virus infection: clinical features, molecular pathogenesis of disease, and latency. Neurol Clin. 2008; 26:675–97. 10.1016/j.ncl.2008.03.011 18657721 PMC2754837

[56] World Health Organization. Varicella and herpes zoster vaccines: WHO position paper. Weekly Epidemiological Record. 2016; 25:265–88.

[57] Schmader K, George LK, Burchett BM, Pieper CF, Hamilton JD. Racial differences in the occurrence of herpes zoster. J Infect Dis. 1995; 171:701–4. 10.1093/infdis/171.3.701 7876622

[58] Hope-Simpson RE. The nature of herpes zoster: a long-term study and a new hypothesis. Proc R Soc Med. 1965; 58:9–20. 10.1177/003591576505800106 14267505 PMC1898279

[59] Schmader K. Herpes zoster in older adults. Clin Infect Dis. 2001; 32:1481–6. 10.1086/320169 11317250

[60] Sharief MK, Hentges R, Ciardi M. Intrathecal immune response in patients with the post-polio syndrome. N Engl J Med. 1991; 325:749–55. 10.1056/NEJM199109123251101 1651456

[61] Julien J, Leparc-Goffart I, Lina B, Fuchs F, Foray S, Janatova I, Aymard M, Kopecka H. Postpolio syndrome: poliovirus persistence is involved in the pathogenesis. J Neurol. 1999; 246:472–6. 10.1007/s004150050386 10431774

[62] Amole M, Khouzam-Skelton N. Diagnosing Post-Polio Syndrome in the Elderly, a Case Report. Geriatrics (Basel). 2017; 2:14. 10.3390/geriatrics2020014 31011024 PMC6371137

[63] Stein A, Purgus R, Olmer M, Raoult D. Brill-Zinsser disease in France. Lancet. 1999; 353:1936. 10.1016/S0140-6736(99)01995-9 10371575

[64] Faucher JF, Socolovschi C, Aubry C, Chirouze C, Hustache-Mathieu L, Raoult D, Hoen B. Brill-Zinsser disease in Moroccan man, France, 2011. Emerg Infect Dis. 2012; 18:171–2. 10.3201/eid1801.111057 22261378 PMC3310116

[65] McQuiston JH, Knights EB, Demartino PJ, Paparello SF, Nicholson WL, Singleton J, Brown CM, Massung RF, Urbanowski JC. Brill-Zinsser disease in a patient following infection with sylvatic epidemic typhus associated with flying squirrels. Clin Infect Dis. 2010; 51:712–5. 10.1086/655891 20687836

[66] Bechah Y, Capo C, Mege JL, Raoult D. Epidemic typhus. Lancet Infect Dis. 2008; 8:417–26. 10.1016/S1473-3099(08)70150-6 18582834

[67] Pai M, Behr MA, Dowdy D, Dheda K, Divangahi M, Boehme CC, Ginsberg A, Swaminathan S, Spigelman M, Getahun H, Menzies D, Raviglione M. Tuberculosis. Nat Rev Dis Primers. 2016; 2:16076. 10.1038/nrdp.2016.76 27784885

[68] Jung RS, Bennion JR, Sorvillo F, Bellomy A. Trends in tuberculosis mortality in the United States, 1990-2006: a population-based case-control study. Public Health Rep. 2010; 125:389–97. 10.1177/003335491012500307 20433033 PMC2848263

[69] Horsburgh CR Jr, O'Donnell M, Chamblee S, Moreland JL, Johnson J, Marsh BJ, Narita M, Johnson LS, von Reyn CF. Revisiting rates of reactivation tuberculosis: a population-based approach. Am J Respir Crit Care Med. 2010; 182:420–5. 10.1164/rccm.200909-1355OC 20395560 PMC2921602

[70] Hochberg NS, Horsburgh CR Jr. Prevention of tuberculosis in older adults in the United States: obstacles and opportunities. Clin Infect Dis. 2013; 56:1240–7. 10.1093/cid/cit027 23362286 PMC3693488

[71] Heidari B. Knee osteoarthritis prevalence, risk factors, pathogenesis and features: Part I. Caspian J Intern Med. 2011; 2:205–12. 24024017 PMC3766936

[72] Englund M, Lohmander LS. Risk factors for symptomatic knee osteoarthritis fifteen to twenty-two years after meniscectomy. Arthritis Rheum. 2004; 50:2811–9. 10.1002/art.20489 15457449

[73] Toivanen AT, Heliövaara M, Impivaara O, Arokoski JP, Knekt P, Lauren H, Kröger H. Obesity, physically demanding work and traumatic knee injury are major risk factors for knee osteoarthritis--a population-based study with a follow-up of 22 years. Rheumatology (Oxford). 2010; 49:308–14. 10.1093/rheumatology/kep388 19946021

[74] Cooper C, Inskip H, Croft P, Campbell L, Smith G, McLaren M, Coggon D. Individual risk factors for hip osteoarthritis: obesity, hip injury, and physical activity. Am J Epidemiol. 1998; 147:516–22. 10.1093/oxfordjournals.aje.a009482 9521177

[75] Coggon D, Reading I, Croft P, McLaren M, Barrett D, Cooper C. Knee osteoarthritis and obesity. Int J Obes Relat Metab Disord. 2001; 25:622–7. 10.1038/sj.ijo.0801585 11360143

[76] Juhakoski R, Heliövaara M, Impivaara O, Kröger H, Knekt P, Lauren H, Arokoski JP. Risk factors for the development of hip osteoarthritis: a population-based prospective study. Rheumatology (Oxford). 2009; 48:83–7. 10.1093/rheumatology/ken427 19056801

[77] Omalu BI, DeKosky ST, Minster RL, Kamboh MI, Hamilton RL, Wecht CH. Chronic traumatic encephalopathy in a National Football League player. Neurosurgery. 2005; 57:128–34. 10.1227/01.neu.0000163407.92769.ed 15987548

[78] Omalu BI, DeKosky ST, Hamilton RL, Minster RL, Kamboh MI, Shakir AM, Wecht CH. Chronic traumatic encephalopathy in a national football league player: part II. Neurosurgery. 2006; 59:1086–92. 10.1227/01.NEU.0000245601.69451.27 17143242

[79] McKee AC, Robinson ME. Military-related traumatic brain injury and neurodegeneration. Alzheimers Dement. 2014; 10:S242–53. 10.1016/j.jalz.2014.04.003 24924675 PMC4255273

[80] Kokjohn TA, Maarouf CL, Daugs ID, Hunter JM, Whiteside CM, Malek-Ahmadi M, Rodriguez E, Kalback W, Jacobson SA, Sabbagh MN, Beach TG, Roher AE. Neurochemical profile of dementia pugilistica. J Neurotrauma. 2013; 30:981–97. 10.1089/neu.2012.2699 23268705 PMC3684215

[81] Nordström A, Nordström P. Traumatic brain injury and the risk of dementia diagnosis: A nationwide cohort study. PLoS Med. 2018; 15:e1002496. 10.1371/journal.pmed.1002496 29381704 PMC5790223

[82] Sun Y, Lee HJ, Yang SC, Chen TF, Lin KN, Lin CC, Wang PN, Tang LY, Chiu MJ. A nationwide survey of mild cognitive impairment and dementia, including very mild dementia, in Taiwan. PLoS One. 2014; 9:e100303. 10.1371/journal.pone.0100303 24940604 PMC4062510

[83] Sullan MJ, Asken BM, Jaffee MS, DeKosky ST, Bauer RM. Glymphatic system disruption as a mediator of brain trauma and chronic traumatic encephalopathy. Neurosci Biobehav Rev. 2018; 84:316–24. 10.1016/j.neubiorev.2017.08.016 28859995

[84] DePinho RA. The age of cancer. Nature. 2000; 408:248–54. 10.1038/35041694 11089982

[85] Niccoli T, Partridge L. Ageing as a risk factor for disease. Curr Biol. 2012; 22:R741–52. 10.1016/j.cub.2012.07.024 22975005

[86] Armitage P, Doll R. The age distribution of cancer and a multi-stage theory of carcinogenesis. Br J Cancer. 1954; 8:1–12. 10.1038/bjc.1954.1 13172380 PMC2007940

[87] Campisi J. Aging, cellular senescence, and cancer. Annu Rev Physiol. 2013; 75:685–705. 10.1146/annurev-physiol-030212-183653 23140366 PMC4166529

[88] van Deursen JM. The role of senescent cells in ageing. Nature. 2014; 509:439–46. 10.1038/nature13193 24848057 PMC4214092

[89] Campisi J. Aging and cancer: the double-edged sword of replicative senescence. J Am Geriatr Soc. 1997; 45:482–8. 10.1111/j.1532-5415.1997.tb05175.x 9100719

[90] Coppé JP, Desprez PY, Krtolica A, Campisi J. The senescence-associated secretory phenotype: the dark side of tumor suppression. Annu Rev Pathol. 2010; 5:99–118. 10.1146/annurev-pathol-121808-102144 20078217 PMC4166495

[91] Baker DJ, Childs BG, Durik M, Wijers ME, Sieben CJ, Zhong J, Saltness RA, Jeganathan KB, Verzosa GC, Pezeshki A, Khazaie K, Miller JD, van Deursen JM. Naturally occurring p16(Ink4a)-positive cells shorten healthy lifespan. Nature. 2016; 530:184–9. 10.1038/nature16932 26840489 PMC4845101

[92] Childs BG, Baker DJ, Wijshake T, Conover CA, Campisi J, van Deursen JM. Senescent intimal foam cells are deleterious at all stages of atherosclerosis. Science. 2016; 354:472–7. 10.1126/science.aaf6659 27789842 PMC5112585

[93] Demaria M, Ohtani N, Youssef SA, Rodier F, Toussaint W, Mitchell JR, Laberge RM, Vijg J, Van Steeg H, Dollé ME, Hoeijmakers JH, de Bruin A, Hara E, Campisi J. An essential role for senescent cells in optimal wound healing through secretion of PDGF-AA. Dev Cell. 2014; 31:722–33. 10.1016/j.devcel.2014.11.012 25499914 PMC4349629

[94] Gems D, Kern CC. Is "cellular senescence" a misnomer? Geroscience. 2022; 44:2461–9. 10.1007/s11357-022-00652-x 36068483 PMC9768054

[95] Wiley CD, Campisi J. The metabolic roots of senescence: mechanisms and opportunities for intervention. Nat Metab. 2021; 3:1290–301. 10.1038/s42255-021-00483-8 34663974 PMC8889622

[96] Sutton M. Late Recurrence of Carcinoma of Breast. Br Med J. 1960; 2:1132–4. 10.1136/bmj.2.5206.1132 20788964 PMC2098195

[97] Omidvari S, Hamedi SH, Mohammadianpanah M, Nasrolahi H, Mosalaei A, Talei A, Ahmadloo N, Ansari M. Very late relapse in breast cancer survivors: a report of 6 cases. Iran J Cancer Prev. 2013; 6:113–7. 25250120 PMC4142915

[98] Dal Lago D, Villa G, Miguoli R, Annoni G, Vergani C. An unusual case of breast cancer relapse after 30 years of disease-free survival. Age Ageing. 1998; 27:649–50. 10.1093/ageing/27.5.649 12675105

[99] Haldane JBS. New Paths in Genetics. London: Allen and Unwin. 1941

[100] Hisama FM, Oshima J, Martin GM. How Research on Human Progeroid and Antigeroid Syndromes Can Contribute to the Longevity Dividend Initiative. Cold Spring Harb Perspect Med. 2016; 6:a025882. 10.1101/cshperspect.a025882 26931459 PMC4817739

[101] Chouraki V, Seshadri S. Genetics of Alzheimer's disease. Adv Genet. 2014; 87:245–94. 10.1016/B978-0-12-800149-3.00005-6 25311924

[102] Diaz de Leon A, Cronkhite JT, Katzenstein AL, Godwin JD, Raghu G, Glazer CS, Rosenblatt RL, Girod CE, Garrity ER, Xing C, Garcia CK. Telomere lengths, pulmonary fibrosis and telomerase (TERT) mutations. PLoS One. 2010; 5:e10680. 10.1371/journal.pone.0010680 20502709 PMC2873288

[103] Schafer MJ, White TA, Iijima K, Haak AJ, Ligresti G, Atkinson EJ, Oberg AL, Birch J, Salmonowicz H, Zhu Y, Mazula DL, Brooks RW, Fuhrmann-Stroissnigg H, et al. Cellular senescence mediates fibrotic pulmonary disease. Nat Commun. 2017; 8:14532. 10.1038/ncomms14532 28230051 PMC5331226

[104] Rutherford SL, Lindquist S. Hsp90 as a capacitor for morphological evolution. Nature. 1998; 396:336–42. 10.1038/24550 9845070

[105] Diguet E, Petit F, Escartin C, Cambon K, Bizat N, Dufour N, Hantraye P, Déglon N, Brouillet E. Normal aging modulates the neurotoxicity of mutant huntingtin. PLoS One. 2009; 4:e4637. 10.1371/journal.pone.0004637 19247483 PMC2645678

[106] Victor MB, Richner M, Olsen HE, Lee SW, Monteys AM, Ma C, Huh CJ, Zhang B, Davidson BL, Yang XW, Yoo AS. Striatal neurons directly converted from Huntington's disease patient fibroblasts recapitulate age-associated disease phenotypes. Nat Neurosci. 2018; 21:341–52. 10.1038/s41593-018-0075-7 29403030 PMC5857213

[107] de Koning AP, Gu W, Castoe TA, Batzer MA, Pollock DD. Repetitive elements may comprise over two-thirds of the human genome. PLoS Genet. 2011; 7:e1002384. 10.1371/journal.pgen.1002384 22144907 PMC3228813

[108] De Cecco M, Criscione SW, Peckham EJ, Hillenmeyer S, Hamm EA, Manivannan J, Peterson AL, Kreiling JA, Neretti N, Sedivy JM. Genomes of replicatively senescent cells undergo global epigenetic changes leading to gene silencing and activation of transposable elements. Aging Cell. 2013; 12:247–56. 10.1111/acel.12047 23360310 PMC3618682

[109] De Cecco M, Criscione SW, Peterson AL, Neretti N, Sedivy JM, Kreiling JA. Transposable elements become active and mobile in the genomes of aging mammalian somatic tissues. Aging (Albany NY). 2013; 5:867–83. 10.18632/aging.100621 24323947 PMC3883704

[110] Gorbunova V, Boeke JD, Helfand SL, Sedivy JM. Human Genomics. Sleeping dogs of the genome. Science. 2014; 346:1187–8. 10.1126/science.aaa3177 25477445 PMC4312280

[111] De Cecco M, Ito T, Petrashen AP, Elias AE, Skvir NJ, Criscione SW, Caligiana A, Brocculi G, Adney EM, Boeke JD, Le O, Beauséjour C, Ambati J, et al. L1 drives IFN in senescent cells and promotes age-associated inflammation. Nature. 2019; 566:73–8. 10.1038/s41586-018-0784-9 30728521 PMC6519963

[112] Zhao Y, Simon M, Seluanov A, Gorbunova V. DNA damage and repair in age-related inflammation. Nat Rev Immunol. 2023; 23:75–89. 10.1038/s41577-022-00751-y 35831609 PMC10106081

[113] Van Meter M, Kashyap M, Rezazadeh S, Geneva AJ, Morello TD, Seluanov A, Gorbunova V. SIRT6 represses LINE1 retrotransposons by ribosylating KAP1 but this repression fails with stress and age. Nat Commun. 2014; 5:5011. 10.1038/ncomms6011 25247314 PMC4185372

[114] Gems D. What is an anti-aging treatment? Exp Gerontol. 2014; 58:14–8. 10.1016/j.exger.2014.07.003 25017442

[115] Eyting M, Xie M, Michalik F, Heß S, Chung S, Geldsetzer P. A natural experiment on the effect of herpes zoster vaccination on dementia. Nature. 2025; 641:438–46. 10.1038/s41586-025-08800-x 40175543 PMC12058522

[116] Harrison DE, Strong R, Sharp ZD, Nelson JF, Astle CM, Flurkey K, Nadon NL, Wilkinson JE, Frenkel K, Carter CS, Pahor M, Javors MA, Fernandez E, Miller RA. Rapamycin fed late in life extends lifespan in genetically heterogeneous mice. Nature. 2009; 460:392–5. 10.1038/nature08221 19587680 PMC2786175

[117] Wilkinson JE, Burmeister L, Brooks SV, Chan CC, Friedline S, Harrison DE, Hejtmancik JF, Nadon N, Strong R, Wood LK, Woodward MA, Miller RA. Rapamycin slows aging in mice. Aging Cell. 2012; 11:675–82. 10.1111/j.1474-9726.2012.00832.x 22587563 PMC3434687

